# Quercetin/Hydroxypropyl-β-Cyclodextrin Inclusion Complex-Loaded Hydrogels for Accelerated Wound Healing

**DOI:** 10.3390/gels8090573

**Published:** 2022-09-08

**Authors:** Nutsarun Wangsawangrung, Chasuda Choipang, Sonthaya Chaiarwut, Pongpol Ekabutr, Orawan Suwantong, Piyachat Chuysinuan, Supanna Techasakul, Pitt Supaphol

**Affiliations:** 1 The Petroleum and Petrochemical College, Chulalongkorn University, Bangkok 10330, Thailand; 2 Research Unit on Herbal Extracts-Infused Advanced Wound Dressing, Chulalongkorn University, Bangkok 10330, Thailand; 3 School of Science, Mae Fah Luang University, Chiang Rai 57100, Thailand; 4 Center of Chemical Innovation for Sustainability (CIS), Mae Fah Luang University, Chiang Rai 57100, Thailand; 5 Laboratory of Organic Synthesis, Chulabhorn Research Institute, Bangkok 10210, Thailand

**Keywords:** quercetin, cyclodextrin, polyvinyl alcohol, inclusion complex, hydrogel

## Abstract

This study concentrated on developing quercetin/cyclodextrin inclusion complex-loaded polyvinyl alcohol (PVA) hydrogel for enhanced stability and solubility. Quercetin was encapsulated in hydroxypropyl-β-cyclodextrin (HP-β-CD) by the solvent evaporation method. The prepared quercetin/HP-β-CD inclusion complex showed 90.50 ± 1.84% encapsulation efficiency (%EE) and 4.67 ± 0.13% loading capacity (%LC), and its successful encapsulation was confirmed by FT-IR and XRD. The quercetin/HP-β-CD inclusion complex was well dispersed in viscous solutions of PVA in various amounts (0.5, 1.0, 1.5. 2.5, and 5.0% *w/v* ratio), and the drug-loaded polymer solution was physically crosslinked by multiple freeze–thaw cycles to form the hydrogel. The cumulative amount of quercetin released from the prepared hydrogels increased with increasing concentrations of the inclusion complex. The introduction of the inclusion complex into the PVA hydrogels had no influence on their swelling ratio, but gelation and compressive strength reduced with increasing inclusion complex concentration. The potential cytotoxicity of quercetin/HP-β-CD inclusion complex hydrogels was evaluated by MTT assay and expressed as % cell viability. The results show biocompatibility toward NCTC 929 clone cells. The inhibitory efficacy was evaluated with 2, 2-diphenyl-1-picrylhydrazyl (DPPH) free radical scavenging assay, and the results show a higher level of antioxidant activity for quercetin/HP-β-CD inclusion complex hydrogels compared with free quercetin. The findings of our study indicate that the developed quercetin/HP-β-CD inclusion complex hydrogels possess the required properties and can be proposed as a quercetin delivery system for wound-healing applications.

## 1. Introduction

At some point, everyone will experience an injury that involves skin or body tissue damage, and it is important to care for it properly. There are many wound care products on the market, and several factors need to be considered when choosing an appropriate dressing for each type of wound, including the color and depth of the wound, degree of infection, amount of exudate, and condition of the peri-wound skin [1]. Normally, an ideal dressing should have the following properties: it should maintain a moist environment, protect from bacterial invasion, accelerate the formation of epithelialization, provide thermal insulation, and be easy to remove without debris after healing [2].

Today, wound dressings have been developed to facilitate biological functions, such as antibacterial, antifungal, and antiviral functions, compared with traditional wound dressings, which just cover wounds. Herbal drugs from medicinal plants are widely incorporated in wound dressing materials to improve the healing properties [3,4]. Quercetin is an interesting component of the flavonoid family that is found in onions, apples, berries, tea, and tomatoes [5,6]. In addition, it has physicochemical features and absorption ability, is a source of dietary nutrition, and principally impacts inflammation and immunological function. Research has shown that ingesting 50 to 800 mg of quercetin per day (quercetin accounts for 75%) may have potential health advantages owing to its easy absorption in the organs responsible for metabolism and elimination [7]. The antioxidant, anti-inflammatory, antibacterial, antiviral, anticancer, antitoxic, and immunomodulatory effects of quercetin prove that it has potential therapeutic value for wound healing.

However, the major problems that limit its biological activity for wound healing are poor solubility, low bioavailability, and hydrophobicity, which means it generally dissolves more readily in organic solution [8,9]. To overcome the disadvantages of quercetin, a drug-delivery system can be used, for example, that forms an inclusion complex with host molecules such as cyclodextrins (CDs), which is one of the most popular techniques [10].

Cyclodextrins, generated by the action of an enzyme on starch, are cyclic oligosaccharides linked by α-1,4 glycosidic bonds that are normally composed of six (α-CD), seven (β-CD), or eight (γ-CD) α-D-glucopyranose units. Because cyclodextrins enhance hydrophobic–hydrophilic interactions between proteins and other molecules, they are employed in the food processing industry to create reduced-cholesterol products and increase the bioavailability of desired molecules [11]. In general, CDs have a cone-shaped structure, with a hydrophilic outer surface and a hydrophobic inner cavity. Due to the hydrophobic cavity, CDs can trap hydrophobic molecules to form host–guest complexes for use in the pharmaceutical industry [12]. CDs are used to encapsulate odors and flavors to avoid food discoloration and minimize off-flavors and can be applied in food packaging [13]. In addition, CDs are used in many medical applications, such as wound dressings. Inclusion complexes containing curcumin and hydroxypropyl–cyclodextrin have been added to bacterial cellulose hydrogels to enhance wound healing [14]. However, mainly hydroxypropyl-β-cyclodextrin (HP-β-CD) is employed as a complexing agent to improve the water solubility and bioavailability of poorly soluble medicines and enhance their stability [15].

Hydrogels are three-dimensional natural or synthetic polymers that contain more than 90% water. The polymer chains are crosslinked to form a network to maintain the hydrogel structure, and the crosslinking process can be either physical or chemical. Hydrogels have a hydrophilic porous structure that can absorb a certain amount of water depending on many factors such as the pore size or the polymer type [16]. Hydrogels are popular for wound dressing because of several benefits including the ability to maintain a moist environment and absorb exudates, good biocompatibility, low adhesion to wound tissue, reduced pressure and shear forces, protection from bacterial invasion, and thermal insulation [16,17]. Hydrogels also have the ability to cool and soothe the skin, which is helpful for pain relief of burns or painful wounds [18]. Polyvinyl alcohol (PVA) is a biodegradable and water-soluble hydrophilic polymer that is produced by hydrolyzing polyvinyl acetate (PVAc). This polymer has good mechanical properties, excellent thermal stability, low toxicity, and high gas barrier properties, so it is widely used in many applications, including biomedical, food packaging, paper coating, and textile sizing and as a thickener and emulsion stabilizer, or it can be blended with other polymers to increase the overall properties. PVA hydrogel wound dressings can be produced by physical crosslinking through the cyclic freeze–thaw process [19]. Due to the PVA structure, they can form H-bonding between −OH groups during freezing, leading to crosslinking of their polymer chains (crystalline zone). However, the freezing conditions, including temperature, time, and number of cycles, could affect the degree of physical crosslinking or crystallinity of the obtained hydrogels. At lower freezing temperature, there are greater interactions between PVA chains and more crystal formation. Small water crystals are formed and sublimated at low freezing temperatures, resulting in a porous structure [20], whereas more freeze–thaw cycles lead to further crystal formation and a higher degree of physical crosslinking [21].

The purpose of this work was to form an inclusion complex by encapsulating quercetin into HP-β-CD to overcome its limitations and to develop new hydrogels incorporating the inclusion complex to accelerate wound healing. Drug-loaded hydrogels were evaluated for their physical properties in terms of gelation, swelling, and compression. The quercetin released from the prepared hydrogels and the cytotoxic effects on an NCTC 929 clone cells were also investigated regarding the biological abilities of the drug-loaded hydrogels.

## 2. Results and Discussion

### 2.1. Characterization of Quercetin/HP-β-CD Inclusion Complex

**Figure 1:**
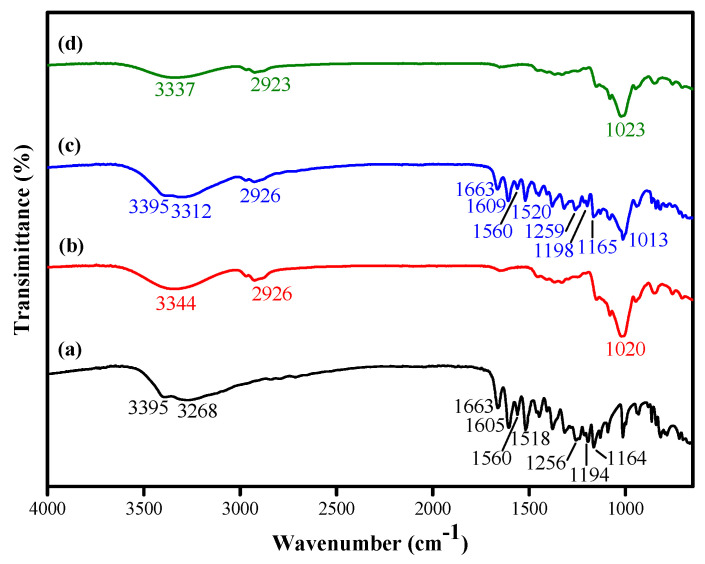
FTIR spectra of (**a**) quercetin, (**b**) HP-β-CD, (**c**) quercetin/HP-β-CD physical mixture, and (**d**) quercetin/HP-β-CD inclusion complex.

FTIR spectroscopy was used to investigate the successful encapsulation of quercetin into HP-β-CD (Figure 1). The FTIR spectrum of quercetin (Figure 1a) showed –OH groups stretching at 3395 and 3268 cm^−1^; C=O aryl ketone stretching at 1663 cm^−1^; C=C aromatic ring stretching at 1605, 1560, and 1518 cm^−1^; and C–O aryl ether ring stretching, C–O phenol stretching, and C–CO–C ketone stretching and bending at 1256, 1194, and 1164 cm^−1^, respectively [22]. For HP-β-CD (Figure 1b), the characteristic broad peak of OH group stretching was shown at 3344 cm^−1^; C–H stretching was detected at 2926 cm^−1^; and C-O-C glucose unit stretching occurred at 1020 cm^−1^ [23]. The FTIR spectrum of the physical mixture of the two (Figure 1c) showed characteristic peaks of quercetin, including C=O aryl ketone stretching (1663 cm^−1^), C=C aromatic ring stretching (1609, 1560, and 1520 cm^−1^), and C–CO–C ketone stretching and bending (1165 cm^−1^), as well as C–O–C glucose unit stretching of HP-β-CD. When quercetin was encapsulated, its characteristic peaks seemed to be masked (Figure 1d). The C=O aryl ketone stretching (1663 cm^−1^), C=C aromatic ring stretching (1605, 1560, and 1518 cm^−1^), and C–CO–C ketone stretching and bending (1164 cm^−1^) peaks of quercetin were absent in the inclusion complex, which may be because the quercetin was entrapped in the HP-β-CD cavities [24]. Signals of the characteristic bands of quercetin shifted from 3282 cm^−1^ to 3142 cm^−1^ O–H bending signals showing the formation of quercetin/HP-β-CD inclusion complex [25].

**Figure 2:**
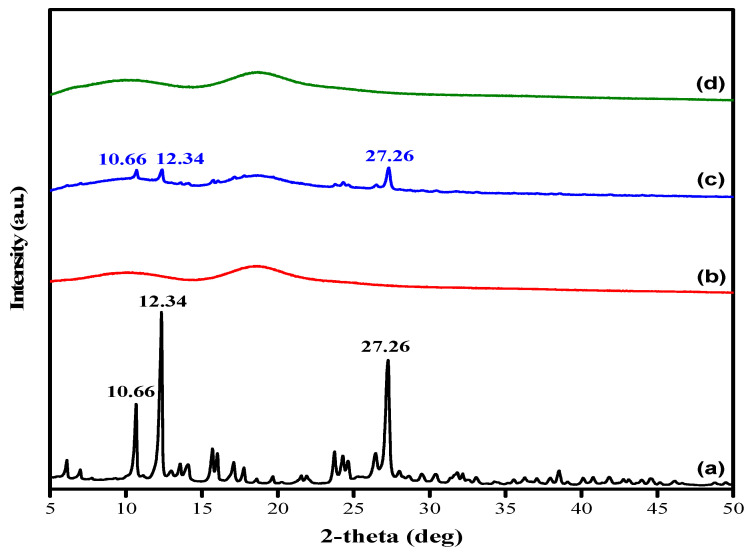
XRD patterns of (**a**) quercetin, (**b**) HP-β-CD, (**c**) quercetin/HP-β-CD physical mixture, and (**d**) quercetin/HP-β-CD inclusion complex.

The XRD patterns provide further evidence of the successful encapsulation of quercetin in HP-β-CD (Figure 2). Characteristic diffraction peaks (Figure 2a) indicated that quercetin existed in a crystalline form, with peaks at three 2θ positions (10.66°, 12.34°, and 27.26°) [26], while HP-β-CD (Figure 2b) showed broad peaks of amorphous materials caused by random X-ray scattering [27]. After the complexation of quercetin and HP-β-CD, there were no noticeable crystalline peaks of quercetin in the inclusion complex (Figure 2d) because the CD cavities hindered its crystalline structure, unlike the physical mixture, which showed crystalline and amorphous behavior (Figure 2c) of quercetin and HP-β-CD, respectively. These results suggest that quercetin was successfully encapsulated into HP-β-CD cavities, leading to the loss of characteristic peaks. Similarly, Zhu et al. encapsulated l-menthol, which is crystalline (2θ = 8.0°, 14.0°, 20.5°, and 21.6°), into amorphous HP-β-CD and observed no sharp peaks of l-menthol [28]. This result agrees with Kim et al., who reported similar XRD patterns of some selective flavonoids in hydroxypropyl-β-cyclodextrin inclusion complexes, which was indicative of the amorphous nature of the complex [29].

**Figure 3:**
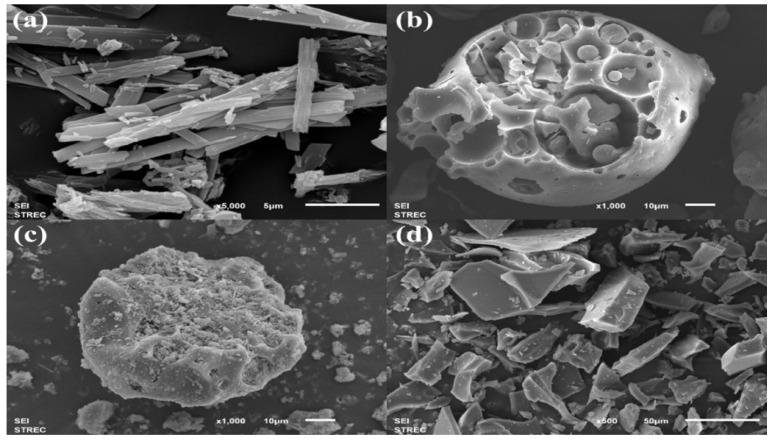
SEM images of (**a**) quercetin, (**b**) HP-β-CD, (**c**) quercetin/HP-β-CD physical mixture, and (**d**) quercetin/HP-β-CD inclusion complex.

The morphologies of quercetin, HP-β-CD, and quercetin/HP-β-CD physical mixture and inclusion complex were observed at different magnifications on SEM (Figure 3). Quercetin (Figure 3a) presented a strip-like structure with a smooth surface [30], while HP-β-CD (Figure 3b) appeared as rough spheres [31]. In the physical mixture (Figure 3c), both quercetin and HP-β-CD kept their own structure, with particles of HP-β-CD embedded with quercetin particles [32]. On the other hand, the morphology and shape of quercetin/HP-β-CD inclusion complex obtained from the solvent evaporation method were completely different from those of the original product (Figure 3d). Our results were consistent with Pradhan et al. [33] in that the morphology of Berberis anthocyanin-loaded CD inclusion complex revealed the rod shape. The encapsulation of quercetin with CD demonstrated the electrostatic and hydrogel bond interactions between quercetin and CD.

### 2.2. Encapsulation Efficiency and Loading Capacity of Quercetin in the Inclusion Complex

Cyclodextrins are nontoxic cyclic oligosaccharides consisting of α1,4-glycosidic bonds. CDs are distinguished by hydrophilic surfaces and hydrophobic cavities. The cavities are suitable for inclusion complexes with hydrophobic drugs (quercetins) to improve the aqueous solubility and yield Van der Waals and hydrophobic forces [34,35]. Quercetin/HP-β-CD inclusion complex was formed with optimum loading and encapsulation efficacy, achieved at a mass ratio of HP-β-CD to quercetin of 4.35:1. Loading capacity (LC) is the ratio of mass of encapsulated compound to mass of the polymer (Equation (1)), and encapsulation efficacy (EE%) refers to the ratio of the mass of the encapsulated compounds to the total mass of the compounds (Equation (2)). Encapsulation is considered to be an excellent carrier of compounds when LC id ≥ 5%, and %EE in the range of 70–100% indicates excellent encapsulation capacity.

The absorbance of ~1 mg/mL inclusion complex in DMSO was 0.68917 ± 0.01715, which can be calculated back to the concentration of quercetin using the predetermined calibration curve (R^2^ = 0.99758). The estimated total quercetin content in the inclusion complex was 0.4554 ± 0.0089 g.

The %EE and %LC of quercetin were quantified with UV-Vis spectrophotometry at a wavelength of 379 nm and calculated using Equations (1) and (2), respectively. The %EE of the obtained quercetin/HP-β-CD inclusion complex was 90.50 ± 1.84%, and the %LC was 4.67 ± 0.13%. The %LC of the inclusion complex seemed to be quite low but was not. The molecular weight of HP-β-CD was greater than that of quercetin by around 4.6 times. We also used the excess molar ratio of HP-β-CD to prepare the inclusion complex. That explains why when we calculated %LC, which compares the drug in the complexation (or inclusion complex) in terms of weight, the molar ratio seemed to be low. This was similar to the results of Hadian et al., who prepared four formulations of geraniol/β-CD inclusion complexes by varying the mole ratio of GR:β-CD (0.44:0.13, 0.44:0.2, 0.44:0.4, and 0.44:1), and showed that %LC gradually decreased with increasing mole ratio (by 7.8 ± 0.70, 6.9 ± 0.1 6.6 ± 0.28 and 6.5 ± 0.6, respectively) [36].

### 2.3. Gelation of PVA Hydrogels Loaded with Quercetin/HP-β-CD Inclusion Complex

The gelation of neat and all prepared drug-loaded hydrogels in PBS (pH = 7.4) at 37 °C for 24 h was calculated using Equation (3). The experiments were repeat five times, and values were averaged. The gelation of neat PVA hydrogel was 97.40 ± 0.62%, while the gelation of drug-loaded hydrogels ranged from 74.52 ± 0.86% to 94.36 ± 0.80%, as shown in Table 1.

The percent gelation of drug-loaded hydrogel clearly gradually decreased with increasing inclusion complex content in the hydrogels. The reduction in hydrogel weight may have occurred for the following reasons. The first is that hydrogel swells when immersed in PBS, leading to a loose structure, so the quercetin can dissolve and be released out of the hydrogels [20]. The second reason is that when loading the quercetin/HP-β-CD inclusion complex into the hydrogels, the inclusion complex may disrupt the crosslink formation of the hydrogel, so some PVA that did not participate in crystallite formation could have dissolved into the solution [21].

### 2.4. Swelling Ratio of PVA Hydrogels Loaded with Quercetin/HP-β-CD Inclusion Complex

**Figure 4:**
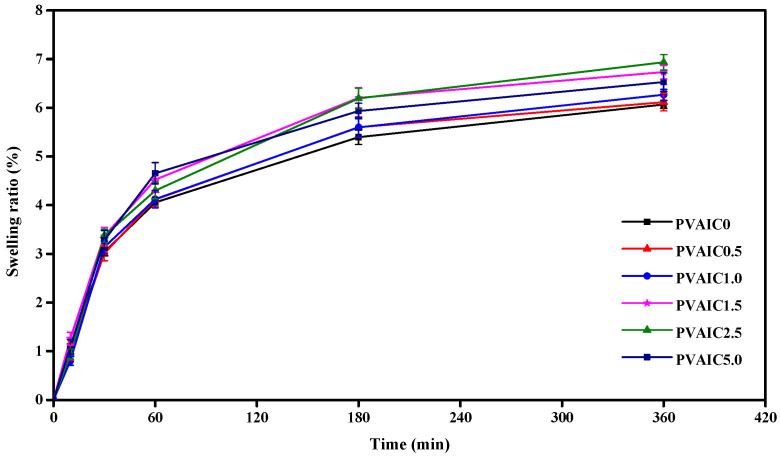
Swelling ratios of PVA hydrogels containing 0% (PVAIC0), 0.5% (PVAIC0.5), 1.0% (PVAIC1.0), 1.5% (PVAIC1.5), 2.5% (PVAIC2.5), and 5.0% (PVAIC5.0) quercetin/HP-β-CD inclusion complex. Data shown as mean ± standard deviation (*n* = 3).

The swelling ratio of neat and drug-loaded hydrogels in PBS (pH = 7.4) at 37 °C at 10, 30, 60, 180, and 360 min was calculated using Equation (4). The experiment was repeated three times at each release time point, and values were averaged. The results show that the swelling ratio of hydrogels with various drug amounts was slightly higher than that of neat PVA hydrogel, but there was not much difference (Figure 4). After the first 60 min of the experiment, the swelling ratio of all hydrogels dramatically increased to around 4.3%, then gradually increased to around 5.8% at 180 min and became relatively stable at around 6.4% after immersion in PBS for 360 min. The incorporation of quercetin/HP-β-CD inclusion complex into the PVA hydrogels had no effect on the swelling ratio, which may be caused by the similar conditions of the freeze–thaw process that we used to prepare the hydrogels. Therefore, the number and temperature of freezing–thawing cycles could be the most important factors controlling the swelling ratio of hydrogels. We can also add other polymers or materials, such as gelatin or chitosan, to increase the swellability of PVA hydrogels [20].

### 2.5. Mechanical Test of PVA Hydrogels Loaded with Quercetin/HP-β-CD Inclusion Complex

The compressive strength of neat and all prepared drug-loaded hydrogels was tested, and the experiment was repeated seven times to obtain average values. The stiffness and compressive modulus of neat PVA hydrogel were 27,318 ± 831 N/m and 1.19 ± 0.07 MPa, respectively, while the values for drug-loaded hydrogels were 19,520 ± 1066 to 26,199 ± 1275 N/m and 0.88 ± 0.04 to 1.12 ± 0.08 MPa, respectively (Table 2). As the amount of inclusion complex in the hydrogels increased, stiffness and compressive modulus seemed to gradually decrease. This may have resulted from the molecules of the inclusion complex being distributed in the PVA structure, leading to a lower crystalline zone [20] and decreased crosslink density of the PVA hydrogel [37], so it was not able to withstand the compressive force compared with lower drug content hydrogels. Water content is another factor that can affect the mechanical properties of hydrogels because when the water in hydrogel evaporates, the hydrogel becomes stronger, so it will have stronger resistance against compressive force than softer or lower-water-content hydrogels [38]. Qing et al. prepared PVA hydrogel dressings that incorporated N-succinyl chitosan (NSCS) and lincomycin, and the results revealed that the introduction of 10% NSCS reduced the compression strength of pure PVA hydrogel from 0.33 to 0.25 MPa because NSCS increases the distance between PVA chains and weakens the molecular interaction force. However, when they further increased NSCS content, hydrogel bonding occurred, and the hydrogel became stronger, leading to enhanced compression strength (0.75 MPa). As NSCS content was further increased to 40 and 50%, the compression strength in turn decreased due to the low crosslinking density resulting from the decreased gel fraction [39].

### 2.6. In Vitro PVA Hydrogels Loaded with Quercetin/HP-β-CD Inclusion Complex Dissolution and Release Kinetics Study

**Figure 5:**
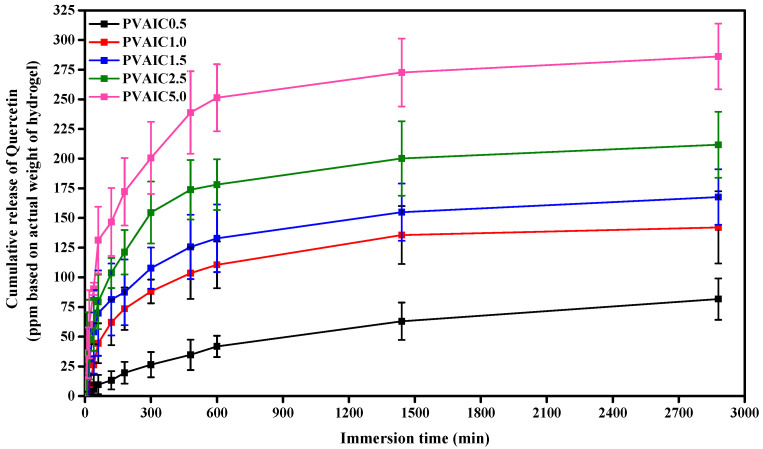
Release profiles of quercetin from PVA hydrogels containing 0.5% (PVAIC0.5), 1.0% (PVAIC1.0), 1.5% (PVAIC1.5), 2.5% (PVAIC2.5), and 5.0% (PVAIC5.0) of quercetin/HP-β-CD inclusion complex. Data shown as mean ± standard deviation (*n* = 3).

The characteristics of quercetin release from the prepared hydrogels were investigated using the total immersion method over a period of 48 h in PBS (pH = 7.4) and incubated at 37 °C under continuous stirring. The cumulative release of quercetin from all hydrogels was calculated using Equation (5) and reported in parts per million. As shown in Figure 5, the cumulative amount of quercetin released from the hydrogels increased rapidly with increasing immersion time for the first 5 or 8 h of the experiment, then gradually increased and reached a plateau. After a period of 48 h, the cumulative quercetin quantities released from the quercetin/HP-β-CD inclusion complex PVA hydrogels were 81.66 ± 17.42, 142.07 ± 30.43, 167.64 ± 23.30, 211.74 ± 27.78, and 286.10 ± 27.71 ppm for PVAIC0.5, PVAIC1.0, PVAIC1.5, PVAIC2.5, and PVAIC5.0, respectively. The greatest cumulative amounts of CIP released were observed when the quercetin concentration of PVAIC5.0 was used, and therefore, it can be concluded that when a greater concentration of quercetin is introduced to the nanoparticles, the drug release will increase. In the USA, the mean quercetin intake was approximately 14.90 to 16.39 mg per day [40]. Thus, the release characteristics of quercetin for the PVAIC5.0 hydrogels (close to 28.6 mg/100 g of sample) was found to be about 1.9-times higher than the oral solution of the quercetin intake per day.

The drug-release kinetics of quercetin from PVA hydrogels loaded with quercetin/HP-β-CD inclusion complex were fitted with Zero-order and Korsmeyer–Peppas models to reveal the kinetics of drug release. Release constant (k) and release exponent (*n*) were calculated to identify the release mechanism, and the fitted model parameters and correlation coefficients (r^2^) for the release profiles are given in Table 3. An r^2^ coefficient of determination over 0.95 was considered to reflect the goodness of fit of the release model [41]. The release data from PVA hydrogels loaded with quercetin/HP-β-CD inclusion complex fit with the Korsmeyer–Peppas model. The release exponent (n) values can be used to indicate the release rate mechanism. The PVAIC0.5, PVAIC1.0, PVAIC1.5, PVAIC2.5, and PVAIC5.0 hydrogels had n values of 1.31, 0.77, 0.68, 0.58, and 0.77, respectively. The n values between 0.89 and 1 indicate that the release of quercetin from PVAIC1.0 to PVAIC5.0 were controlled by the mechanism of anomalous (non-Fickian) diffusion. The diffusion exponent for PVAIC0.5 is in the range of 0.89 and 1, which denotes the case II transport mechanism (zero-order kinetics).

### 2.7. In Vitro Antioxidant Activity: DPPH-Radical Scavenging Ability Assay

**Figure 6:**
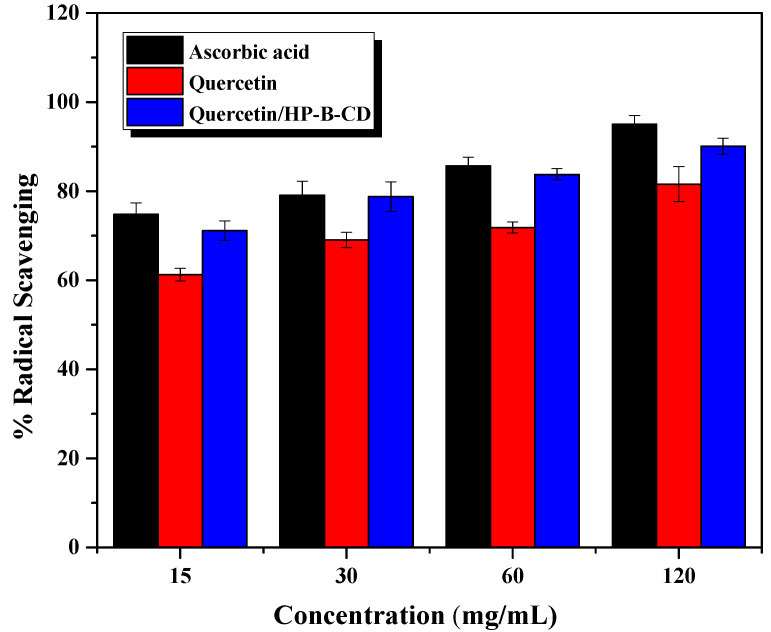
Scavenging activity of ascorbic acid, quercetin and quercetin/ HP-β-CD as determined by DPPH assay. Values expressed as mean ± standard deviation (*n* = 5).

The encapsulation of quercetin within cyclodextrin increases the antioxidant activity compared with free molecules [42]. The antioxidant activity of quercetin/HP-β-CD inclusion complex is widely used in 1,1-diphenyl-2-picrylhydrazyl (DPPH) radical scavenging assay in comparison with free counterparts and the results were shown in Figure 6. A concentration of 120 µg/mL quercetin/HP-β-CD inclusion complex was determined to have 74.80% DPPH inhibition compared with free quercetin, with 61.23% of inhibition, and ascorbic acid with 74.80%. HP-β-CD inclusion complex showed significant antioxidant activity compared with quercetin alone (Figure 6) in a dose-dependent manner. The results indicate that HP-β-CD inclusion complex is associated with the sustained release of quercetin and increases the stability of incorporated bioactive compounds such as quercetin, significantly improving its antioxidant activity [43].

### 2.8. Cytotoxicity Evaluation

**Figure 7:**
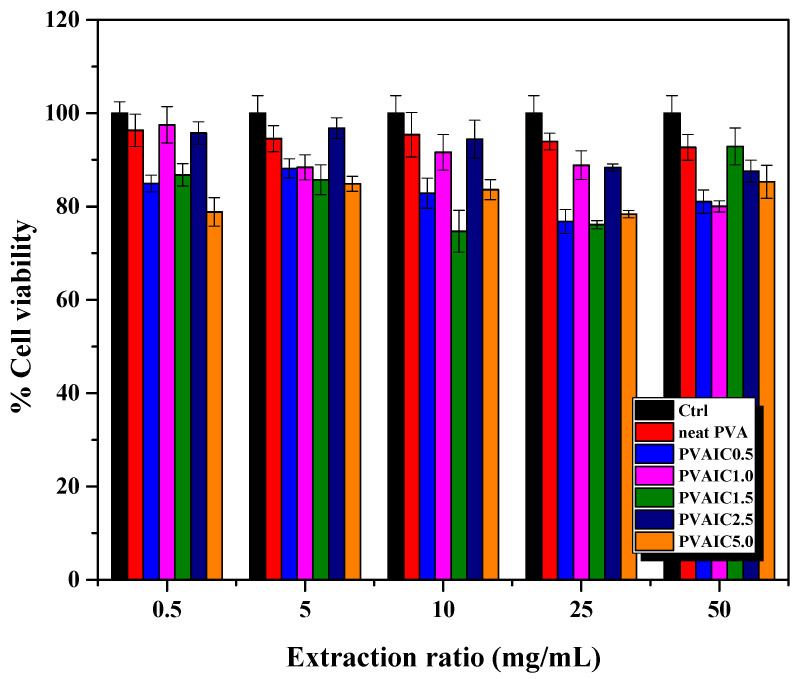
Indirect cytotoxicity of PVA hydrogels loaded with various amounts of quercetin/HP-β-CD inclusion complex with extraction medium: neat PVA, 0.5, 5, 10, 25, and 50 mg/mL cultured with NCTC 929 clone cells.

The indirect cytotoxicity evaluation of all samples toward NCTC 929 clone cells investigated the potential use of drug loaded PVA hydrogels as wound dressing material. Figure 7 shows the viability of cells obtained from MTT assay after being cultured with various concentrations of media extracted from neat PVA hydrogel as blank PVA, and PVA hydrogels loaded with quercetin/HP-β-CD inclusion complex (PVAIC0.5, PVAIC1.0, PVAIC1.5, PVAIC2.5, and PVAIC5.0) compared with that obtained after cells were cultured with fresh SFM. According to the in vitro cytotoxicity standard, a reduction of cell viability by more than 30% is considered a cytotoxic effect [44]. The viability of NCTC 929 clone cells with all extraction medium concentrations, i.e., neat PVA, PVAIC0.5, PVAIC1.0, PVAIC1.5, PVAIC2.5, and PVAIC5.0, were 92.7–96.3, 76.8–88.2, 80.0–97.5, 74.7–92.9, 87.6–96.8, and 78.3–85.3%, respectively. Cell viability was higher than 70% in all prepared hydrogels loaded with quercetin/HP-β-CD inclusion complex. Therefore, all hydrogels loaded with quercetin/HP-β-CD inclusion complex with extraction medium concentrations of 0.5, 5, 10, 25, and 50 mg/mL had cell viability of more than 70%, indicating that these conditions were non-cytotoxic, and the hydrogels have potential in wound dressing applications.

## 3. Conclusions

We successfully prepared PVA hydrogels containing quercetin/HP-β-CD inclusion complex that achieved efficient drug loading and effective encapsulation. The quercetin/HP-β-CD inclusion complex was prepared with an excess molar ratio of HP-β-CD via the solvent evaporation method. The successful encapsulation of quercetin in HP-β-CD was confirmed by FT-IR and XRD, in which characteristic peaks of quercetin could not be observed after encapsulation due to the hindering of CD cavities. As observed on SEM, the quercetin/HP-β-CD inclusion complex demonstrated rod-shaped particles. The prepared quercetin/HP-β-CD inclusion complex had 90.50 ± 1.84% encapsulation efficiency and 4.67 ± 0.13% loading capacity. It was incorporated into PVA hydrogels in various amounts (0, 0.5, 1.0, 1.5, 2.5, and 5.0% MIC/V_solvent ratio_). The hydrogels were evaluated for their potential use as wound dressing in terms of gelation, swelling, compression, release characteristics, antioxidant properties, and in vitro non-cytotoxicity. The incorporation of quercetin/HP-β-CD inclusion complex into PVA hydrogels had no effect on the swelling ratio of the hydrogels. The gelation and compressive strength of hydrogels decreased with increasing inclusion complex content, whereas the cumulative release of quercetin from hydrogels increased with higher amounts of inclusion complex.

Hydrogels loaded with quercetin/HP-β-CD inclusion complex investigated for their cytotoxicity in vitro by MTT assay were shown to be nontoxic toward mouse fibroblast NCTC 929 clone cells at the test concentrations. The results showed significantly increased antioxidant properties of quercetin/HP-β-CD inclusion complex compared with free quercetin. These findings suggest that the use of quercetin/HP-β-CD inclusion complex ensures non-cytotoxicity and potential antioxidant activity. Hence, hydrogel with quercetin/HP-β-CD inclusion complex is an attractive candidate for the encapsulation of bioactive compounds for biomedical applications.

## 4. Materials and Methods

### 4.1. Materials

Quercetin hydrate (C_15_H_10_O_7_·xH_2_O, Mw 302.24, >96% purity) and hydroxypropyl-β-cyclodextrin (HP-β-CD, Mw ~1380–1500, >99.0% purity) were purchased from Tokyo Chemical Industry (Portland, OR, USA). Polyvinyl alcohol (PVA; Mw 89,000–98,000, 99+% hydrolyzed) was purchased from Sigma Aldrich (Saint Louis, MO, USA). Methanol (MeOH; HPLC grade) and dimethyl sulfoxide (DMSO; AR grade) were purchased from RCI Labscan (Bangkok, Thailand).

### 4.2. Formation of Quercetin/HP-β-CD Inclusion Complex

Lyophilized formulations of quercetin/HP-β-CD inclusion complex were prepared by freeze-drying using the neutralization method [45]. Freeze-drying is an excellent method for preserving a wide variety of heat-sensitive materials such as proteins, microbes, pharmaceutical agents, tissues, and plasma [46]. The molar ratio of quercetin to HP-β-CD, following Lan et al., was 1:4.35 [24]. First, 10 g of HP-β-CD was dissolved in 50 mL distilled water, and 0.5 g of quercetin was dissolved in 70 mL MeOH at 60 °C to obtain a homogeneous solution. Then, both solutions were mixed and refluxed at 65 °C overnight. The solution was stirred continuously at room temperature for 5 h for complete encapsulation. Next, MeOH was removed by heating the solution at 75 °C for 2 h. The solution was freeze-dried after being filtered (0.45 μm) and frozen at −20 °C. Finally, quercetin/HP-β-CD inclusion complex was obtained after freeze-drying at −50 °C for 48 h. The resulting lyophilized quercetin/HP-β-CD inclusion complex powder was stored in airtight containers and protected from light until use. Freeze-dried drug–cyclodextrin complexes were used for hydrogel preparation.

### 4.3. Characterization of Quercetin/HP-β-CD Inclusion Complex

Fourier-transform infrared spectrometry (FT-IR; Nicolet iS5 with iD7, Thermo Scientific, Waltham, MA, USA) was performed to reveal the chemical structure and group interaction of inclusion complex and analyzed in ATR mode with a framework region of 650 to 4000 cm^−1^ with 64 scans at resolution of 4 cm^−1^. Samples were prepared as KBr pellets.

The crystalline structure of quercetin/HP-β-CD inclusion complex was examined by X-ray diffractometry (XRD; SmartLab, Rigaku, Japan) with diffraction angle of 2θ ranging from 5 to 50° at a scanning rate of 0.02°/s.

A scanning electron microscope (SEM; JEOL, JSM-6610LV, Japan) was used to observe the morphology of quercetin/HP-β-CD inclusion complex with 15 kV accelerating voltage and magnification of 5000, 1000, and 500 times after the samples were vacuum-coated with a fine layer of gold.

### 4.4. Determination of Encapsulation Efficiency and Loading Capacity of Quercetin/HP-β-CD Inclusion Complex

The drug-loading process was carried out through a diffusional mechanism, and the encapsulation efficiency (%EE) was used to illustrate the quantity of added drug encapsulated in the formulation. The amount of encapsulated quercetin was determined using a direct method [18] and recorded on a calibration curve at 379 nm.

Briefly, 20 mg of quercetin/HP-β-CD inclusion complex was dissolved in 20 mL DMSO. The amount of quercetin was quantified using a UV-Vis spectrophotometer at a wavelength that provided the maximum absorbance (379 nm), and the concentration was back-calculated from the predetermined quercetin calibration curve. Encapsulation efficiency (%EE) and loading capacity (%LC) were calculated from the following equations [47]:

(1) $$EE\;(\%)\;=\;\frac{W_{Entrapped\;\;Quercetin}}{W_{Quercetin\;\;loaded}}\times \;100$$

(2) $$LC\;(\%)\;=\;\frac{W_{Entrapped\;\;Quercetin}}{W_{Sample}}\times \;100$$

### 4.5. Preparation of Quercetin/HP-β-CD Inclusion Complex-Loaded Hydrogels

Quercetin/HP-β-CD inclusion complex was loaded into 10% *w/v* PVA hydrogels. Briefly, 2 g of PVA was slowly dissolved in 20 mL distilled water at 80–100 °C for 40 min. Then, an accurate weight of quercetin/HP-β-CD inclusion complex was added to PVA solutions at concentrations of 0, 0.5, 1.0, 1.5, 2.5, and 5.0% *w/v*, which were labeled as conditions PVAIC0, PVAIC0.5, PVAIC1.0, PVAIC1.5, PVAIC2.5, and PVAIC5.0, respectively. Then, the solutions were stirred continuously for 20 min to obtain homogeneous solutions with uniform distribution. The homogeneous solutions were then sonicated in an 80 °C ultrasonic bath to remove air bubbles. Then, the viscous solutions were poured into 90 mm Petri dishes and covered with aluminum foil. The drug-loaded PVA hydrogels were physically crosslinked by repeated freeze–thaw cycles, with freezing at −20 °C for 20 h then thawing at room temperature for 4 h [48]. This freeze–thaw process was repeated for 4 cycles to obtain quercetin/HP-β-CD inclusion complex-loaded hydrogels. The Petri dishes containing prepared hydrogels were then sealed with parafilm and kept in the refrigerator.

### 4.6. Gelation of PVA Hydrogels Loaded with Quercetin/HP-β-CD Inclusion Complex

To investigate the gel fractions of the hydrogels used in the study, it was first necessary to ensure that the samples had achieved a constant weight. The prepared hydrogels were cut into 0.5 cm radius round and then dried in an oven at 50 °C for 48 h to obtain the dry weight (W_dry_). After that, the dried hydrogels were submerged in phosphate-buffered saline (PBS, pH = 7.4) at 37 °C for 24 h with agitation (100 rpm) and then were dried again at 50 °C for 48 h to obtain the hydrogel weight after extraction (W_after extraction_):

(3) $$Gelation\;(\%)\;=\;\frac{W_{after\;\;extraction}}{W_{dry}}\times \;100$$

### 4.7. Swelling Ratio of PVA Hydrogels Loaded with Quercetin/HP-β-CD Inclusion Complex

The swelling ratio was measured by taking the dried hydrogel (m_dry_) and the wet mass (m_wet_) of the hydrogel after immersion in a solution for a specified time [49]. First, each hydrogel was cut into circles of 0.5 cm radius that were then immersed in 5 mL of phosphate-buffered saline (PBS, pH = 7.4) at 37 °C with agitation (100 rpm) for 10, 30, 60, 180, and 360 min. Finally, the swelling ratio of the hydrogels was calculated using the following equation:

(4) $$Swelling\;ratio\;(\%)\;=\;\frac{m_{wet}-m_{dry}}{m_{dry}}\times \;100$$

### 4.8. Mechanical Test of PVA Hydrogels Loaded with Quercetin/HP-β-CD Inclusion Complex

To observe the effect of loading the drug into the hydrogel on its mechanical properties, compression testing was performed. The hydrogels were cut into circles of 0.5 cm radius in all experiments. The compressive strength of neat PVA hydrogel and drug-loaded hydrogels was measured with a universal testing machine (LRX-Plus, AMETEK Lloyd Instruments Ltd., Hampshire, UK) in terms of stiffness and compressive modulus. The loading cell was set at a 500 N, with 0.05 N preload and 1 mm/min speed, and the measurement was stopped when the load reached 10 N [50].

### 4.9. In Vitro PVA Hydrogels Loaded with Quercetin/HP-β-CD Inclusion Complex Dissolution and Release Kinetics Study

The cumulative amount of released quercetin/HP-β-CD from hydrogels was measured using an immersion method according to Chuysinuan et al. [51] with minor modifications. The disc-shaped specimens (2.8 cm in diameter) were immersed in phosphate-buffered saline (PBS, pH = 7.4) and incubated at 37 °C under continuous stirring. The sample solution (1 mL) was withdrawn at specific time intervals, and an equal amount of fresh release medium was added. The absorbance of released quercetin was measured with a UV-Vis spectrophotometer (LAMBDA 850+, PerkinElmer, Waltham, MA, USA) at a wavelength of 370 nm. The obtained data were back calculated from the predetermined quercetin calibration curve (R^2^ = 0.9944) to determine the amounts of quercetin released from the hydrogel samples. The cumulative release of quercetin from each hydrogel at different time points (with measurement carried out in triplicate at each time point) was reported as parts per million of the amounts of quercetin released (W_Quercetin released_) divided by the weight of hydrogels (W_hydrogel_):

(5) $$Cumulative\;\;release\;(\%)\;=\;\frac{W_{Quercetin\;\;released}}{W_{hydrogel}}\times \;10^{6}$$

A release kinetics study was used to ascertain the release mechanism, and the quercetin release data were fitted to Korsmeyer–Peppas and zero-order models. The first 60% of the drug release data was used to fit the models.

The Korsmeyer–Peppas model is a simple model that describes drug release from polymer nanoparticle systems [52,53]:

M_t_/M_∞_ = kt^n^

where M_t_/M_∞_ is the quercetin release fraction at time t, k is a constant incorporating geometric structural features, and n is the release exponent indicating the release rate mechanism. The value of n indicates the mechanism of the release; a value around 0.45 indicates case I (Fickian) diffusion, between 0.45 and 0.89 indicates anomalous (non-Fickian) diffusion, and between 0.89 and 1 indicates case II transport (zero-order kinetics).

A zero-order kinetic model is used to describe drugs that are released slowly with a constant concentration and can be characterized as ideal kinetic model in that it maintains constant drug levels during the delivery process [54,55]:

M_t_/M_∞_ = kt

where M_t_/M_∞_ is the quercetin release fraction at time t, with zero-order rate constant, and t is investigation time.

### 4.10. In Vitro Antioxidant Characterization

The antioxidant activity of free quercetin and quercetin/HP-β-CD inclusion complex was evaluated using 2, 2-diphenyl-1-picrylhydrazyl (DPPH) free radical scavenging assay following [3]. First, quercetin and quercetin/HP-β-CD in various concentrations (15, 30, 60, and 120 µg/mL) were dissolved in methanol. Then, the samples (1 mL) were mixed with 3 mL DPPH solution (0.1 mM in methanol). The mixture was incubated for 30 min in the dark. Finally, the absorbance of the reaction mixture was measured using a microplate reader at 517 nm. Radical scavenging activity (%) was calculated using the following equation. Ascorbic acid was used as a standard and all analyses were performed in triplicate:

(6) $$DPPH\;radical\;scavenging\;activity\;(\%)\;=\;\frac{\left(Absorbance\;of\;control\;-\;Absorbance\;of\;tested\;sample\right)}{Absorbance\;of\;control}\times \;100$$

### 4.11. Cytotoxicity Evaluation

While CDs are chosen for pharmaceutical formulations to increase the solubility, bioavailability, and stability of many medications, their structure and cytotoxic capability are crucial for more effective drug delivery [56]. To evaluate the potential biomedical applications of PVA hydrogels loaded with various amounts of quercetin/HP-β-CD inclusion complex, their biocompatibility in terms of indirect cytotoxicity toward NCTC 929 clone cells (ATCC-CCL-1, Rockville, MD, USA (17th passage) was evaluated in accordance with the ISO10993-5 standard test method [57]. First, cells were cultured in Dulbecco’s Modified Eagle Medium (DMEM (1×); GIBCO, Waltham, MA, USA) containing 10% fetal bovine serum (FBS; GIBCO, USA) and 1% antibiotic–antimycotic agent (GIBCO, Waltham, MA, USA). Then, the cells were seeded into 96-well tissue-culture polystyrene (TCPS) plates (SPL Lifescience, Pochon, Korea) at 8000 cells/well and then incubated at 37 °C in a humidified atmosphere containing 95% air and 5% CO_2_. Next, the hydrogel samples were sterilized by exposure to UV radiation for 30 min/side and were immersed in serum-free medium (SFM, containing DMEM and 1% antibiotic–antimycotic agent) in a 96-well TCPS plate. The samples were incubated for 24 h to produce sample extraction at five ratios of extraction medium (0.5, 5, 10, 25, and 50 mg/mL). NCTC 929 clone cells were cultured separately in culture medium in wells of TCPS plates at 8000 cells/well for 24 h to allow cells to attach to the well surface. After that, the medium was replaced with an extraction medium, and mouse fibroblast L929 cells were incubated for a further 24 h.

The viability of cells cultured in each extraction medium was determined with 3-(4,5-dimethylthiazol-2-yl)-2,5-diphenyltetrazolium bromide (MTT) assay. After treatment, the medium was removed, and the samples were washed with PBS; then, MTT solution (0.5 mg/mL) was added, and samples were incubated for 3 h. After that, the MTT solution was removed from the well and replaced by DMSO (Labscan, Bangkok, Thailand) to dissolve the formazan crystals. Finally, the absorbance of the solutions was measured at 570 nm using a microplate reader (BioTek Instruments, Winooski, VT, USA) to investigate cell viability. The viability of cells cultured with fresh SFM was used as a control.

## Figures and Tables

**Table 1:** Gelation value and mass content of PVA hydrogels containing various amounts of quercetin/HP-β-CD inclusion complex. Data shown as mean ± standard deviation (*n* = 5). IC as quercetin/HP-β-CD inclusion complex.

Sample	Gelation (%)	Mass Content (%)	
		IC	PVA
PVAIC0	97.40 ± 0.62	0.00	100.00
PVAIC0.5	94.36 ± 0.80	4.67	95.24
PVAIC1.0	92.29 ± 0.71	9.09	90.91
PVAIC1.5	89.88 ± 0.83	13.04	86.96
PVAIC2.5	85.59 ± 0.95	20.00	80.00
PVAIC5.0	74.52 ± 0.86	33.33	66.67

**Table 2:** Mechanical test of PVA hydrogels containing various amounts of quercetin/HP-β-CD inclusion complex. Data shown as mean ± standard deviation (*n* = 7).

Sample	Stiffness (N/m)	Compressive Modulus (MPa)
PVAIC0	27,318 ± 831	1.19 ± 0.07
PVAIC0.5	26,199 ± 1275	1.12 ± 0.08
PVAIC1.0	24,878 ± 758	1.09 ± 0.10
PVAIC1.5	24,011 ± 941	0.99 ± 0.06
PVAIC2.5	21,817 ± 1001	0.92 ± 0.04
PVAIC5.0	19,520 ± 1066	0.88 ± 0.04

**Table 3:** Modeling results of quercetin released from PVA hydrogels loaded with quercetin/HP-β-CD inclusion complex fitting with Zero-order and Korsmeyer–Peppas models.

Sample	Release Kinetic Models			
	Zero-Order		Korsmeyer–Peppas	
	k	r^2^	*n*	r^2^
PVAIC0.5	0.78	0.7953	1.31	0.995
PVAIC1.0	0.67	0.9127	0.77	0.9671
PVAIC1.5	1.10	0.844	0.68	0.9526
PVAIC2.5	1.49	0.7033	0.58	0.9748
PVAIC5.0	2.31	0.6468	0.77	0.9535

## Data Availability

Not applicable.

## Abbreviations

CDs	Cyclodextrins
DMSO	Dimethyl sulfoxide
DPPH2	2-diphenyl-1-picrylhydrazyl
EE	Encapsulation efficiency
FT-IR	Fourier-transform infrared spectrometry
GR	Geraniol
HP-β-CD	Hydroxypropyl-β-cyclodextrin
IC	Quercetin/HP-β-CD inclusion complex
LC	Loading capacity
m	Mass
MeOH	Methanol
MTT	3-[4,5-dimethylthiazol-2-yl]-2,5 diphenyl tetrazolium bromide
NCTC 929	Connective mouse tissue (L cell, L-929, derivative of Strain L)
NSCS	N–succinyl chitosan
PBS	Phosphate-buffered saline
PVA	Polyvinyl alcohol
PVAc	Polyvinyl acetate
SEM	Scanning electron microscope
TCPS	Tissue-culture polystyrene
W	Weight
XRD	X-ray powder diffraction
